# Preventable Mortality in Regions of Slovakia—Quantification of Regional Disparities and Investigation of the Impact of Environmental Factors

**DOI:** 10.3390/ijerph16081382

**Published:** 2019-04-17

**Authors:** Beata Gavurova, Peter Toth

**Affiliations:** 1 Center for Applied Economic Research, Faculty of Management and Economics, Tomas Bata University in Zlín, 760 01 Zlín, Czech Republic; 2 Faculty of Economics, Technical University of Košice, 040 01 Košice, Slovak Republic; peter.toth@tuke.sk

**Keywords:** preventable mortality, regional disparities, environmental factors, prevention programs

## Abstract

Environmental health is among the priority areas of public health, and the current professional communities are intensively engaged with it. The main objective of the study is to quantify regional disparities of preventable mortality in Slovakia and to study the extent of the influence of selected environmental factors on changes in the development of its values. A cross-sectional linear regression model is used to quantify effects of environmental factors on the preventable mortality. Also, cluster analysis is used to identify regions with similar levels of air pollution. Environmental factors were selected based on the study of the World Health Organization. From the point of view of the influence of environmental factors on preventable mortality in the case of men, statistically significant connection to sewerage, SO_2_ production, and production of particulate matter was demonstrated. In the case of women, equally important factors showed connection to sewerage and SO_2_. The results of this study point to significant regional disparities in preventable mortality and a different degree of impact of environmental factors. Preventable mortality is above the Slovak average in most of the least-developed districts. Even in this group, there are significant differences.

## 1. Introduction

Both scientific and professional communities constantly discuss the issue of measuring the performance and efficiency of health systems. None of the concepts developed recently are final. The concepts lack complex observations of performance and efficiency of health systems without any restrictions. The most widely used concept in recent decades is the concept of avoidable (amenable and preventable) mortality. This concept measures the effectiveness of the health system by quantifying premature deaths that could be avoided by proper health interventions. It is also an important concept in terms of international comparisons of health outcomes in individual countries. The examination of its potential has been the subject of many foreign studies. International scientific studies were conducted mainly within the European Union (EU) member states (e.g., Treurniet et al. [1], Gavurova and Vagasova [2], Weber and Clerc [3], Van den Heuvel et al. [4]), OECD countries (Gay et al. [5], Gianino et al. [6]) and other selected European countries (Korda and Butler [7], Stirbu et al. [8], Nolte and McKee [9], Plug et al. [10]). The concept of avoidable mortality is also considered as a suitable indicator of country development assessment. Many authors focus only on one component of avoidable mortality, either preventable or amenable. For instance, Van den Heuvel et al. [4] especially focuses on preventable mortality in their study as the most significant issue in European countries. Also, the authors examined environmental indicators and their impact on the development of preventable mortality, besides linking preventable mortality to health expenses. The quantification of regional disparities in mortality within a particular country or among countries is frequently present in many studies when examining the development of avoidable mortality. Factors such as socio-economic status, education, social deprivation of the population, and environmental factors are of interest to many research teams. The importance of examining the environmental determinants of population health has increased in recent years. Nearly one and a half million people die in the European region each year from environmentally-related diseases according to World Health Organization (WHO) data. This represents 16% of the total deaths and the total burden of diseases caused by environmental risks that may be avoided or eliminated. The European Environment Agency report, called “Unequal exposure and unequal impacts: social vulnerability to air pollution, noise, and extreme temperatures in Europe”, highlights a close connection between social and environmental issues in Europe. The expansion of these environmental threats and the effects they have on human health also reflects differences in income levels, unemployment, and education. According to this report, up to 86% of premature deaths in Europe are the result of diseases that are directly or indirectly related to the environment in which that person lives. The most well-known health impacts are connected to air pollution, poor water quality, and hygiene. According to the World Health Organization report “Global Health Risks” [11], the environment has a significant impact on human health in many ways—by exposure to various physical, chemical, and biological risk factors. Major environmental risk factors include the following: unsafe drinking water sources, hygiene and personal hygiene habits, external air pollution (especially in the cities), and air pollution due to combustion of solid fuels. Similar to other studies, this study points to the differences in the effects of environmental factors among countries, but also within countries and geographically limited areas, which are affected by factors other than those currently investigated. 

These consistent facts emphasize the importance of continual examination of the development of preventable mortality in individual countries. The above-mentioned focus also highlights a discovery of new factors that affect this process at the national and international level. Motivation for a realization of this study in the Slovak conditions emerged from all of the above-mentioned facts. The main objective of the study is to quantify regional disparities in the development of preventable mortality and to examine the extent of the impact of selected environmental factors on changes in the development of its values. The outputs of the study represent a valuable platform for national and regional health policy makers, as well as for the creation of an international comparison platform.

### Literature Review

Numerous research studies from the last decades have dealt with the issue of regional health disparities in different geographical locations. In some studies, the authors compare different countries, while other studies focus on geographical levels within a particular country, which form a basis of comparative analysis. The spectrum of examined determinants that influence the development of mortality differs in various studies. Kontopantelis et al. [12] examined regional disparities in the mortality of the population aged 25 to 44 at the geographical level, focusing on the north and south of England. Authors examined whether the increase in age-specific mortality is geographically differentiated, and if so, which factors cause these differences. The results of the study show that the overall mortality rate of the population for cardiovascular disease has declined during the period under review, but the differences between the north and the south of the country in the mortality of these diseases persist. The main cause is the deterioration of existing social and health inequalities. Deaths were higher in the male group than in females throughout the time span. The primary reason of geographical inequalities in mortality was the use of drugs, alcohol consumption, and cardiovascular diseases. These reasons represent two-thirds of the excessive mortality, one third of which is the inequality caused by cultural differences. Minton and McCartney [13] criticize the above-mentioned study by Kontopantelis et al. [12], arguing that significant regional disparities in mortality are not between the north and south, but between London and the rest of the territory. The authors criticize the geographical breakdown of the territory to the north and south of England for the purposes of these analyses and consider it as inappropriate for any research of the differences in mortality. The authors state that the city of London has the lowest mortality rate. On the other hand, in other urban areas of England, higher mortality rates were determined rather than in rural settlements. Similarly, the authors criticize the age structure of the studied population. The age range of 25–44 years should be more disaggregated, which would allow a better understanding and limitation of mortality risks in different age categories. 

Benett et al. [14] chose the disparities in mortality the period 2002–2016 for their analysis, which used data on mortality from the Office for National Statistics broken down by gender and 5-year age groups. The team also used the index of multiple deprivation. According to the results of their analysis, the expected life expectancy at birth in 2016 was lower in poorer areas (from 78.8 years to 86.7 years for women, and from 74 to 83.8 years for men). The age-standardized mortality rate caused by multiple diseases and injuries was higher in more vulnerable groups. Hematological disorders, and prostate and breast cancer represent exceptions. The largest disparities were noticed among the most and the least vulnerable groups (in 2016) in cases of deaths caused by ischemic heart disease, respiratory diseases, lung cancer, and dementia. Relative inequality was higher for lung cancer, diabetes, and respiratory diseases. The highest inequalities in life expectancy were caused by the mortality of children of less than 5 years. The significance of mortality on ischemic heart disease decreased for both genders, and the significance of the respiratory system increased. In conclusion, the authors claim that smoking, alcohol consumption, and unbalanced diet, which are also the causes of social inequalities, also represent the main causes of many diseases. Therefore, the determinants of inequalities in the expected life expectancy are important. 

Steel et al. [15] analyzed data from 1990 to 2016, geographically geared to the following areas: England, Scotland, Wales, Northern Ireland (i.e., United Kingdom) and 150 higher territorial units in England. The main subject of the analysis is as follows: 264 causes of GBD (Global Burden of Disease) deaths, 20 specific types of deaths, and 84 types of risk or risk clusters. The leading causes of YLL (Years of Life Lost) have been characterized by ischemic heart disease, oncological lung disease, cerebrovascular disease, and chronic obstructive pulmonary disease. The results of their study focus on regional disparities in mortality development, as well as factors that affect it. Authors point to a lack of data related to the main reasons of poor health at the local level. Consequently, the possibility of detecting new factors determining the development of mortality due to demographic aging processes in individual localities was lost. The concept of demographic aging also represents the basic research pattern of analysis in the study by Hanlon et al. [16]. The aim of their study was to investigate the relationship between frailty, multimorbidity, specific long-term conditions, and the mortality of the middle and older age population. The analysis used UK Biobank data and applied multinomial logistic regression that compared sociodemographic characteristics and long-term conditions of weakness of participants. Models were developed to explore the relationship between sociodemographic characteristics and weakness and their impact on mortality. 

Dicker et al. [17] investigated the relationship between age-specific mortality and its development through a sociodemographic index (education, income, and fertility, aged under 25). The authors examined the development of mortality rates in 1950–2017 in 23 age categories, across both genders, and in 918 geographically limited territories. Macroscopic analysis focused on 195 countries. Public data and household surveys were used. According to the authors, mortality and life expectancy are two indicators that record heterogeneous trends and differences in trends between men and women over a longer period of time. These trends were most significant in the period between 1950 and 2005, and since 2005 these disparities have gradually narrowed. In 2017, women in Central Europe, Eastern Europe, and Central Asia had expected life expectancy higher than men, by 9.1 years. The second highest difference was recorded in Latin America and the Caribbean, where the median expected life expectancy of women was up to 6.1 years higher than that of men. 

Lozano et al. [18] linked health assessment to sustainable development goals (SDG) to map sustainable development of countries. The aim of the study was to examine the values of the SDG indicators with new indicators: health workers’ density, sexual violence, population status after census, and prevalence of physical and sexual violence. The study included 195 countries, and the period of 1990–2017 was analyzed. As the results of the study show, most countries are expected to have higher SDG in 2030 than in 2017. Western European countries, Canada, Japan, and Singapore are among the countries with the best SDG scores. Afghanistan and sub-Saharan Africa showed the worst values. In terms of gender, worse health results were evident in men rather than in women—higher mortality and risk. The authors emphasize that without paying more attention to NCD (non-communicable disease), many countries will not be able to meet the SDG’s goals. The impact of environmental risk factors on preventable mortality was studied by Van den Heuvel et al. [4]. These authors looked at several variables, and the results of their study showed that air pollution had no significant impact on preventable mortality. Pautrel [19] and Ebenstein et al. [20] also looked at the impact of air pollution in the country on health outcomes, with the resultant indicator being the life expectancy in these studies. Pautrel [19] points to the fact that there is a space for improvement in health condition through a stricter environmental policy of the country and offers several solutions. Ebenstein et al. [20] states that communicable disease mortality rates are very likely to be responsive to hospital construction and public health initiatives, but less sensitive to pollution. High pollution exposure is associated with declines in life expectancy, but this effect would not be judged as statistically significant by conventional criteria. There is, however, a statistically significant relationship between pollution exposure and higher rates of cardiorespiratory mortality. Both studies highlight the need to further explore the links between the environment, health, and economic activity in the country. Effects of negative impacts of environmental factors on population health have been reflected for decades, and according to the authors, have implications for social security funding and intergenerational redistribution.

These studies are highly heterogeneous, determined primarily by the research objectives of specific research teams. Their research structure shows a wide variability of the factors examined in relation to mortality and its regional differences. They offer valuable inspiration for deeper exploration and detection of other health determinants that are not visible in a comprehensive health assessment at the aggregate level. This is important for setting effective prevention programs aimed at eliminating the emergence of civilization diseases that have a very unfavorable social and economic impact on society in each country. Underestimating their research may have negative impacts on the sustainability of the health and social system in the country in the future.

## 2. Materials and Methods 

The main aim of the paper is to identify the fundamental environmental risk factors that cause preventable mortality in Slovakia, which can be a basis for government programs focused on the decrease of preventable mortality. According to the Act No. 336/2015, the districts in Slovakia are divided into 2 groups: the least-developed districts with high unemployment rates, and other districts. In this analysis, identification of the least-developed districts based on the unemployment rate is studied. It is considered as an appropriate approach for extra government expenditure in order to minimize regional disparities in preventable mortality in Slovakia.

Cross-sectional linear regression was used to identify factors that affect preventable mortality. The Slovak Republic data were used to measure these relationships. The Slovak Republic is heterogeneous country in many aspects. Mainly, socio-economic disparities are significant. There are several government activities aimed at eliminating these differences. Therefore, the government has identified the least-developed districts. Based on the Act No. 336/2015, the least-developed district are districts with an unemployment rate 1.5 times higher than the average unemployment rate in the Slovak Republic for at least nine calendar quarters during the previous twelve follow-on calendar quarters. The government supports these districts by special programs. At present, there are 20 districts included in the list of the least-developed districts from all of the 79 districts in the Slovak Republic. All of them are situated in the Eastern part and the Southern part of the country. This includes the following regions: Lučenec district (LC), Poltár district (PT), Revúca district (RA), Rimavská Sobota district (RS), Veľký Krtíš district (VK), Kežmarok district (KK), Sabinov dsitrict (SB), Svidník district (SK), Vranov nad Topľou district (VT), Gelnica district (GL), Rožňava district (RV), Sobrance district (SO), Trebišov district (TV), Bardejov district (BJ), Medzilaborce district (ML), Košice-okolie district (KS), Levoča district (LE), Snina district (SV), Stropkov district (SP), and Michalovce district (MI). District labels are applied from the promulgation of the Statistical Office of the Slovak Republic No. 597/2002.

This analysis uses data from several sources. The list of the least-developed districts comes from the Central Office of Labour, Social Affairs, and Family. The dataset of the mortality in the Slovak Republic is provided by the National Health Information Centre of the Slovak Republic. The data of percentage of household connected to the water supply and sanitary sewer in the Slovak districts are from the Water Research Institute of the Slovak Republic. The fourth source of data is the Statistical Office of the Slovak Republic. Two datasets from this source were used. The population data consists the average population in each district and data about the air pollution. The dataset of the mortality consists of all deaths in the Slovak Republic. Each deceased person has following attributes: year, district, region, age, gender, and death diagnosis. In the case of air pollution, the following were considered: nitrogen oxides (NO_x_), sulphur dioxide (SO_2_), particulate matter (PM), and carbon monoxide (CO). All air pollutants are expressed as a production in tons. All data are available for the year 2015.

The research is divided into two parts. The first part examines the impact of selected indicators on preventable mortality using cross-sectional linear regression. In the second part, the cluster analysis was applied to identify districts with similar attributes that may be used as a platform for the government action plans to increase the life conditions in these districts. According to the European Commission, preventable mortality includes all deaths that could have been avoided by public health interventions, focusing on factors such as behaviour, lifestyle, socio-economic factors, and environmental factors. The list of causes of deaths included under preventable mortality is given by the Office for National Statistics [21]. That list is adopted by the European Commission, too. There are eight types of causes of deaths: Infections; Neoplasms; Nutritional, endocrine, and metabolic diseases; drug use disorders; cardiovascular diseases; respiratory disorders; unintentional injuries; and intentional injuries.

Mathematically, the preventable mortality is calculated as the sum of all standardized death rates (*SDR*) for specified causes and age categories. Curtin and Klein [22] and Anderson and Rosenberg [23] define *SDR* by Equation (1), where *x* is age category 0; 1–4; 5–9; …; 90–95; 95+, *m_ix_* represents age-specific death rate, *ESP* represents European Standard Population established by the European Commission [24], and index *i* denotes district.

(1) $$SDR_{i}=\frac{\sum_{x}m_{ix}ESP_{x}}{\sum_{x}ESP_{x}}100,000$$

Age specific death rate *m_ix_* is given by Equation (2), where *D_x_* represents number of deceased persons in age category *x* in district *i*, and *P_ix_* is average population in age category *x* in district *i*.

(2) $$m_{ix}=\frac{D_{ix}}{P_{ix}}$$

Preventable mortality (*PMR*) in district *i* is calculated by Equation (3), where *j* is from 1,2, …, *n* and *n* denote the number of diagnoses included in the list of the preventable causes of deaths.

(3) $$PMR_{i}=\underset{j}{\sum }SDR_{ij}$$

*PMR* is applied in the cross-sectional linear model as a dependent variable, which is employed to measure the impact of specified factors on the *PMR*. The general linear regression model for the district *i* can be expressed by Equation (4), where *y* is the dependent variable, *x* represents independent variables, *ε* denotes error term, and *β* is parameter which expresses the effect of a given independent variable [25].

(4) $$y_{i}=\beta_{0}+\beta_{1}x_{i1}+\cdots +\beta_{k}x_{ik}+\cdots +\beta_{K}x_{iK}+\varepsilon_{i}$$

Ordinary least squares estimation (OLS) is based on assumptions that are necessary to verify by statistical tests. Null hypothesis of homoscedasticity was tested by Breusch-Pagan test. Multicollinearity is tested by Variance Inflation Factors. Normality of residuals is tested by Jarque-Bera test, which tests the null hypothesis of zero skewness and sharpness of residuals distribution. 

In the given model, the relationship between preventable mortality and environmental factors is examined. The aim of the model is to quantify the relation between quality of environment and preventable mortality. The following environmental factors were considered: percentage of household connected to the water supply (WS), percentage of households connected to the sanitary sewer (SS), nitrogen oxides (NO_x_), sulphur dioxide (SO_2_), particulate matter (PM), and carbon monoxide (CO). Cross-sectional linear regression for the year 2015 was provided. According to the World Health Organization [26], an environment significantly affects public health in many points, such as influence of different physical, chemical, and biological risk factors. The riskiest environmental factors are unsafe sources of drinking water, hygiene and personal hygienic habits, external air pollution, mainly in large cities, and air pollution due to combustion of solid fuels. In the analysis, models for both sexes are considered separately.

The dummy variable Least-developed, which may inhabit two values, zero and one, is included in this analysis. If the district belongs to the least-developed regions according to unemployment rate, the dummy variable is equal to 1, if it is not, the dummy variable is 0. That division is realized by the Central Office of Labor, Social Affairs, and Family. This variable was added in order to find out whether the list of the least-developed districts is appropriate to prepare special government programs focused on the increase of the quality of life in these districts. When that dummy variable will not be statistically significant, it will mean that it is necessary to identify new measurement that will be a more suitable platform for government programs.

The second step of the study is aimed at identification of districts with similar environmental conditions that could be used as a base for special programmes prepared by the government. It is assumed that the least-developed districts should not be measured only by the unemployment rate, rather there are other factors that measure quality of life. This paper primarily focuses on environmental indicators. To reach that aim, cluster analysis is applied. One of the most widely-used methods based on minimizing a formal objective function is k-means clustering [27]. Let *X* represent a finite set of data items, *X* = {*x*_1_, …, *x_n_*} and each data item is given by a vector *v_x_*. Then, *K* may be non-empty disjoint subsets *C* = {*c*_1_, …, *c_K_*}, and *n*(*c*) is the number of data items referred to in cluster *c*. The minimized objective function is given by Equation (5), where *d*(·,·) denotes distortion measure; in the analysis the Euclidean distance is applied and *v_c_* is the centroid vector calculated by Equation (6) ([25,28]).

(5) $$D\left(C\right)≝\frac{1}{n}\underset{c}{\sum }\underset{x\epsilon c}{\sum }d\left(v_{x},v_{c}\right)$$

(6) $$v_{c}≝\frac{1}{n\left(c\right)}\underset{x\epsilon c}{\sum }v_{x}$$

The heuristic Hartigan-Wong [29] algorithm was used. The advantage of that algorithm is better optimization than standard Lloyd’s method [30]. 

It is recommended to realize several random starts to attain local optimality. There were 20 random starts set. The key task is to set the optimal number of clusters. The optimal number of clusters according to five methods are determined. Silhouette is a graphical method that displays objects located within the cluster and objects between clusters [31]. Duda-Hart Index compares the number of data items referred to in the cluster n(c) with critical value [32]. Milligan-Cooper Index minimalizes the value of absolute second differences between levels of the index [33]. The Ball-Hall Index maximizes difference between hierarchy levels of the index [34]. The last applied method is McClain-Rao Index [35], which minimizes the value of the index. 

## 3. Results

**Figure 1:**
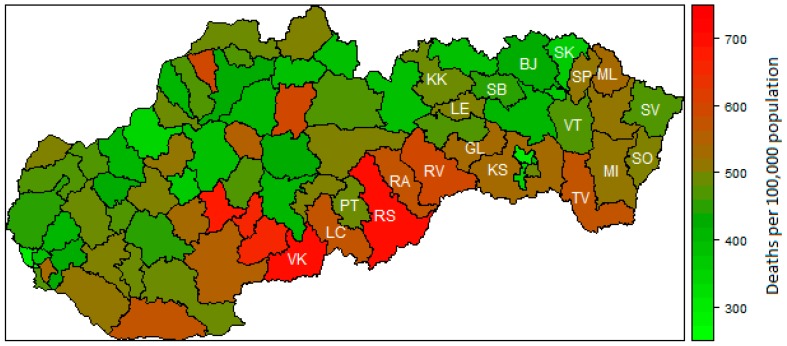
Preventable mortality of men in 2015. Note: Lučenec district (LC), Poltár district (PT), Revúca district (RA), Rimavská Sobota district (RS), Veľký Krtíš district (VK), Kežmarok district (KK), Sabinov dsitrict (SB), Svidník district (SK), Vranov nad Topľou district (VT), Gelnica district (GL), Rožňava district (RV), Sobrance district (SO), Trebišov district (TV), Bardejov district (BJ), Medzilaborce district (ML), Košice–okolie district (KS), Levoča district (LE), Snina district (SV), Stropkov district (SP), and Michalovce district (MI).

**Figure 2:**
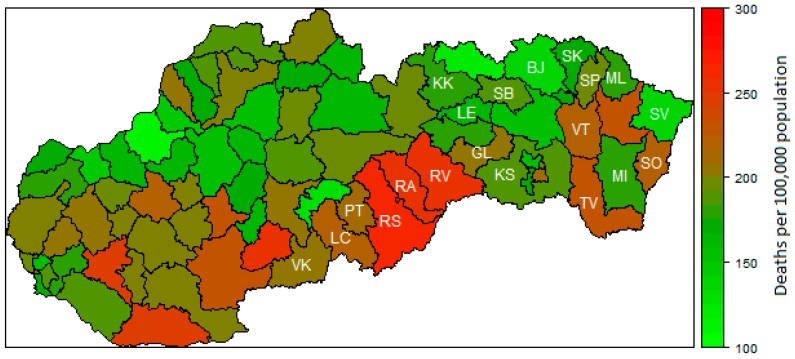
Preventable mortality of women in 2015. Note: Lučenec district (LC), Poltár district (PT), Revúca district (RA), Rimavská Sobota district (RS), Veľký Krtíš district (VK), Kežmarok district (KK), Sabinov dsitrict (SB), Svidník district (SK), Vranov nad Topľou district (VT), Gelnica district (GL), Rožňava district (RV), Sobrance district (SO), Trebišov district (TV), Bardejov district (BJ), Medzilaborce district (ML), Košice–okolie district (KS), Levoča district (LE), Snina district (SV), Stropkov district (SP), and Michalovce district (MI).

Firstly, a descriptive statistic of used data is provided. The average preventable mortality (PMR) of men is 485 deaths per 100,000 inhabitants. The lowest PMR of men is 283 deaths per 100,000 inhabitants in Bratislava I district. On the other hand, the highest PMR of men is 719 deaths per 100,000 inhabitants in Rimavská Sobota district. In general, PMR of women is markedly lower than PMR of men; the average value of PMR for women is 192. The lowest PMR value is 108 in Trenčín district and the highest value is 273 in Rimavská Sobota district, which is the district with the highest PMR of men, too. In most of the least-developed regions, the preventable mortality of men is above the national average. In other words, the highest preventable mortality of men is in Rimavská Sobota district, Veľký Krtíš district, and Rožňava district. The least-developed districts in the North-Eastern part of the Slovak Republic do not suffer as much from the preventable mortality of men. The preventable mortality of men in Stropkov district, Bardejov district, and Sabinov district are significantly lower than the national average. The preventable mortality of men in 2015 in the Slovak districts is depicted in Figure 1. The highest preventable mortality of women is in the least-developed districts Rimavská Sobota district, Revúca district, and Rožňava district. The preventable mortality of women in the North-Eastern districts is lower than the average in the Slovak Republic. It is the same in the case of men. For all districts, the preventable mortality of women is depicted in Figure 2.

On average 88% households are connected to the water supply. The highest connection is almost 100% in Martin district followed by districts in the largest cities in the Slovak Republic. The lowest share of households connected to the water supply is 88% in Bytča district. Generally, the lowest connection of households to the water supply is in the Southern and Eastern part of the Slovak Republic. The connection to the sanitary sewers is lower than connection to the water supply. The highest percentage of households connected to the sanitary sewer is in the Bratislava city, the capital of the Slovak Republic. There are almost 99% households connected to the sanitary sewer. The second highest connection is in the second largest city, Košice. On the contrary, only 30% of households are connected to the sanitary sewer in the Bytča district. About 33% of households are connected in the Námestovo district, Košice-okolie district, and Trebišov district. Average connection to the sanitary sewer in the Slovak Republic is 64% of households. 

In the case of air pollution, there are three districts with massive production of pollutants. The first one is Košice city (contains four individual districts), with production of carbon monoxide at 113,000 tons. Also, the production of other gasses is high. Production of PM is more than 3000 tons, production of Sulphur dioxide is almost 8500 tons, and production of nitrogen oxides reaches 7800 tons. That enormous production of air pollutants is caused by the steel-mill located in the city. Production of all pollutants is significantly higher than in the capital city Bratislava, the largest city in the Slovak Republic. The second highest polluted district is Prievidza district, with production of Sulphur dioxide at more than 46,000 tons. Production of nitrogen oxides is 4160 tons, production of PM is 1440 tons, and carbon monoxide production is about 1600 tons. There is a thermal power station in that district, which is one of the highest polluters in the region. That power station burns brown coal from nearby mines. The third district is Žiar nad Hronom district, with an aluminium-producing company. Consequently, there is over 15,000 tons of carbon monoxide and about 1800 tons of Sulphur dioxide produced in the district. Production of other pollutants is about the average of the Slovak Republic.

In general, the least polluted districts are in the North-Eastern part of the country. The lowest production of nitrogen oxides is 39,700 tons in the Stropkov district. On the other hand, the highest production is 7820 tons in the Košice city. The average production of nitrogen oxide in the Slovak Republic is 898 tons. A similar situation is seen in the case of Sulphur dioxide, which is again produced the least in the North-Eastern part of the Slovak Republic. That is confirmed by the fact that the lowest production of Sulphur dioxide is in the Stropkov district. The largest production of Sulphur dioxide is in the Prievidza district. The average production in the Slovak Republic is 1290 tons. Distribution of PM is relatively uniform across the country. In addition to Košice city, there are other districts in the Southern and Northern part of the country with high PM production. The highest production is 3000 tons in Košice city, in contrast with the 107 tons in the Senec district. The mean production of the PM in the Slovak Republic is 578 tons. The last studied pollutant is carbon monoxide, with an average production in the Slovak Republic of 6720 tons. The highest production is in Košice city followed by the Žiar nad Hronom district. The third highest production of carbon monoxide is in the Trenčín district. The least-polluted districts are situated in the North-Eastern part of the country, and also the majority of districts in the Western part belong to the unpolluted regions in the case of carbon monoxide. The lowest production of carbon monoxide is again in the Senec region. More detailed descriptive statistics are presented in Table 1. When the air pollution is considered from the point of view of the least-developed districts, two groups may be identified. The first one contains districts in the North-East of the country. These districts belong to districts with clean air. The second group of districts from the Southern part is more polluted, which is due to heavy industry being located in these districts, as well as geographical conditions. There are valleys with low scattering conditions which often lead to warnings of air pollution in these districts.

### Impact of Environmental Factors on the Preventable Mortality

Based on the presented methodology, the cross-sectional linear model was applied, with preventable mortality as the dependent variable and seven explanatory variables. Six of them belong to environmental factors in addition to a dummy variable, which expresses the development of the district based on the unemployment rate. Two models were provided, one for each sex. According to the Variance Inflation Factors (VIF), variable NO_x_ was eliminated from the models in order to prevent the existence of the multicollinearity. In the case of NO_x_, the VIF was above 15, and based on the study by Kutner et al. [36], VIF higher than 10 means high multicollinearity. Estimated regression coefficients for both sexes are presented in Table 2.

In the case of men, there are three factors that statistically significantly affect the preventable mortality. If the percentage of households connected to the sanitary sewer rise by one percent point, the preventable mortality rate for men decreases by 2.88 deaths per 100,000 inhabitants. In the area of air pollution, production of SO_2_ and production of PM are statistically significant. The increase in production of SO_2_ by one unit leads to decline of the preventable mortality of men by 0.004 deaths per 100,000 inhabitants. The increment in production of PM by one unit causes the growth of the preventable mortality of men by 0.072 deaths per 100,000 inhabitants. Based on the model, the preventable mortality of women is affected by two factors: percentage of households connected to the sanitary sewer and the production of SO_2_. Signs of both estimated coefficients are the same as in the model for men. The increase in the percentage of households connected to the sanitary sewer by one percent point causes the decrease in the preventable mortality of women by 0.72 deaths per 100,000 inhabitants. The increase of SO_2_ production induces the reduction of the preventable mortality of women by 0.001 deaths per 100,000 inhabitants, which is the same result as in the case of men. It is assumed that this fact occurs due to the outliers, such as the Prievidza district with huge production, which may negatively influence the estimation. 

When the variable Least-developed is considered, this variable is not statistically significant in both models. It may be admitted that the institute of the least-developed districts based on the unemployment rate is not suitable for special prevention programs making. Therefore, cluster analysis was realized in order to identify districts with similar levels of risk factors, for which special prevention programs should be made. In the first step, while doing k-means clustering, it is necessary to set the number of clusters. There are many methods of how to do it. Five of them were applied. The optimal number of clusters according to these methods, as well as values of the indices, are presented in Table 3. All of them suggest three clusters.

**Figure 3:**
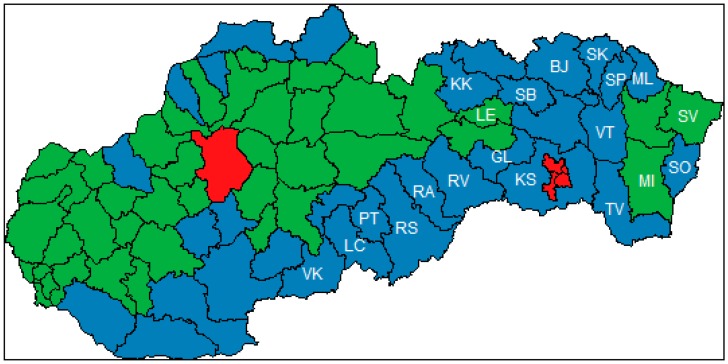
Cluster analysis of Slovak districts. Note: Colors represent clusters of districts, Lučenec district (LC), Poltár district (PT), Revúca district (RA), Rimavská Sobota district (RS), Veľký Krtíš district (VK), Kežmarok district (KK), Sabinov dsitrict (SB), Svidník district (SK), Vranov nad Topľou district (VT), Gelnica district (GL), Rožňava district (RV), Sobrance district (SO), Trebišov district (TV), Bardejov district (BJ), Medzilaborce district (ML), Košice-okolie district (KS), Levoča district (LE), Snina district (SV), Stropkov district (SP), and Michalovce district (MI).

Three clusters of districts in the Slovak Republic were identified. Centroids of these clusters are shown in Table 4. The first cluster, called “Red”, is typical for a high percentage of households connected to the water supply and sanitary sewers, and extremely huge production of all air pollutants. There are only two districts in the cluster: Košice city (consists of four independent districts but due to the data availability we take it as one district in the analysis) and Prievidza district. These districts are specific because of the companies located there. They are depicted in red in Figure 3. The second cluster is called “Green”, and districts from that cluster are filled in green in Figure 3. The average district in that cluster has about 93 percent of households connected to the water supply, while the percentage of households connected to the sanitary sewer is nearly 70 percent. In the case of air pollution, production of NO_x_ is 439 tons, production of SO_2_ is 228 tons, production of PM is 362 tons, and production of CO is 1220 tons. In conclusion, there are districts with high connection rates to the water and sanitary infrastructure and high production of air pollutants in the “Green” cluster. This is the result of industry location in these districts. The last cluster is called “Blue”, and is filled in blue in Figure 3. As it is presented in Table 4, the average values of variables for these districts are relatively low. The percentage of households connected to the water supply is almost 80 percent and percentage of households connected to the sanitary sewer is lower than 50 percent. The production of NO_x_ is 271 tons, production of SO_2_ is 112 tons, production of PM is 548 tons, and production of CO is 841 tons. These districts are less developed, with low connection to the water infrastructure and low production of air pollutants because there is a lack of industry, which leads to higher unemployment and poverty. Figure 3 shows almost all of the least-developed districts according to unemployment rate belong to that cluster (the least-developed districts according to the unemployment rate are labelled by the district mark). There are also several districts from the Northern and South-Western part of the Slovak Republic included in the cluster.

Provided cluster analysis showed that there are three significant groups of districts in the Slovak Republic. That fact should be considered by government institutions responsible for special policies. The unemployment rate does not provide enough information about regional disparities in the Slovak Republic. From the point of view of preventable mortality and quality of life, there are other districts to which the government should focus.

## 4. Discussion

The results of the analyses clearly confirm the existence of regional health disparities in the individual districts of Slovakia. Differences are apparent not only in the horizontal geographical line, but also in the vertical. Significant differences between the East and West of the country, as well as between individual districts belonging to the group of the least-developed regions and those of others, are confirmed. The lowest preventable mortality in men was found in Bratislava I at 290, the highest in the Rimavská Sobota district at 720. Prevalent mortality is above the Slovakian average in most of the least-developed districts, with the exception of districts in the North-Eastern part of Slovakia. Also, in this group of the least-developed districts of Slovakia, there are obvious differences: in the North-Eastern part of these districts, the mortality rate is relatively low (Bardejov district, Stropkov district, and Sabinov district) and represents about 400 deaths per 100,000 inhabitants for men. Gender differentiation also has a significant impact on different results of the surveyed indicators. For women, the highest preventable mortality was recorded in the least-developed districts in the northeast of Slovakia in Rimavská Sobota district, Revúca district, and Rožňava district, with more than 250 deaths per 100,000 inhabitants. This study was also focused on the research of selected factors influencing preventive mortality, with the following factors being selected: Water and Sewer Connections, SO_2_ Production, and PM. 

In the case of men, a statistically significant connection to sewerage, SO_2_, and PM was demonstrated. In the case of women, equally important factors were shown, namely connection to sewerage and SO_2_. An interesting view was also provided by the cluster analysis, which aimed to point to the related areas according to the results of the analysis and the input values of the studied factors. 

The findings provide valuable information for further investigation of the evolution of the indicators’ values, as well as the factors that act on them. Detailed summary of the results unambiguously points to the fact that there have always been significant disparities among some regions of Slovakia. These are partly determined by the structure of the region, its industrial excellence, competitiveness, demographic and socio-economic characteristics, and so on. A very significant factor is the average income in the surveyed area, as well as the migration of the labor force, which also acts to change the values of preventable mortality and regional health disparities. Also, the availability of health care in Slovak regions does not reflect the exact health needs of the population in the regions or the demographic composition of the population. This makes it difficult to set up processes to meet the health needs of the population in selected localities. These factors also depend on the degree of prevention, their structure, and localization, which are important factors in examining the indicators of preventable hospitalizations (to what extent neglected outpatient healthcare has caused hospitalization health care). It is understandable that inpatient healthcare is much costlier to the health system than outpatient healthcare, which is even more challenging for patients and for their quality of his life. The availability of preventative health care initiatives is currently at an insufficient level in Slovakia. The Slovak republic has not yet developed concepts of prevention programs that would be linked to regional characteristics, to retrospective morbidity development, reflection on sociodemographic factors, environmental factors of the locality in which they live, etc. This is due to insufficiently implemented health analyses, which should be the basis for their development and mapping of their basic determinants. High-quality analysis for effective health policies requires permanent work with accurate, up-to-date data, and it is important to create additional databases and interconnect them to eliminate separate policy-making of multiple sectors. For instance, there is narrow penetration in the interconnection of health and social policy, labor market policy, and so on. The database in Slovakia is limited by the collection of data under Slovak legislation, which makes the analytical processes and international comparisons more difficult. In addition to data issues for the development of effective policies, it is also important to use methods and methodologies that highlight the quality and effectiveness of the healthcare system. These are also very important for national and international benchmarking.

The government has divided districts in Slovakia into two groups based on unemployment rate. Districts with high unemployment rate are categorized as the least-developed, and the government allocates extra money to reduce regional disparities. The study results show that division of districts in Slovakia based on unemployment rate alone is not appropriate in the field of the healthcare and preventable mortality. That findings provide knowledge for policy makers, showing that it is necessary to include other factors to classify districts that need special care in the case of health policy. The weakness of the analysis is in the data availability, because data for only one year were used. For further research, it is necessary to collect data systematically, which provides information about development over time. In the next research study, it would be apt to include other variables, mainly socio-economic factors, which also seemingly affect the preventable mortality.

## 5. Conclusions

In order to create effective policies in the health system, it is essential to use quality methodologies and implement specific analyses with an emphasis on regional health disparities. There is a wide range of factors, which are very demanding and dependent on quality databases at the lowest geographic levels. This represents an issue in several countries. Relevance of the methodologies for the creation of effective health policies in Slovakia by applying the concept of preventable mortality and examining the factors influencing its development and changes were emphasized. The main objective of the study was to quantify regional disparities of preventable mortality in Slovakia and to study the extent of the influence of selected environmental factors on changes in the development of its values. The study focused on the least-developed regions, which are identified by the unemployment rate, to study regional disparities. The calculations and construction of preventable mortality is not easy, which discourages experts from continually using them in the development of strategic national and regional plans. Examination of environmental factors in the context of health indicators represents an important issue related to the creation of quality concepts to support the health of the Slovak population and the development of effective policies. These are also desirable in assessing the quality of life of the population. This study points out the importance of these analytical links, as in recent years there have been foreign studies declaring the impact of the environment and factors related to population health. Although their impact is not visible in the short term, its underestimation results in the design of prevention programs without considering these important facts, which will bring many undesirable economic and social impacts in the future. The study results point to significant regional disparities in preventable mortality and the extent of the impact of environmental factors have on it. The differences are apparent not only between the western and eastern parts of the country, but also within the regions between the districts and the group of least-developed districts. Preventable mortality was above average in most of the least-developed districts of Slovakia. Even in this group of the least-developed districts of Slovakia, there were clearly visible differences: in the North-Eastern part of these districts, the preventable mortality was relatively low. Gender differentiation has also a significant effect on different results. In the case of women, the highest preventable mortality is lower than the lowest preventable mortality of men. From the point of view of the influence of environmental factors on males, statistically significant factors are connection to sewerage, SO_2_ production, and PM. In the case of women, important factors were connection to sewerage and SO_2_. The study results declare the fact that this geographical structure is not significant in relationships between preventable mortality and environmental factors. The results of this study will help health and social policy makers develop national and regional development plans, as well as create a strategic framework for the Slovak Republic. There results of the study may contribute to supporting the creation of a high-quality data base enabling the investigation of a wide range of factors affecting the health of the population and the preparation of quality local prevention programs. This will help to eliminate regional health disparities and improve the values of avoidable deaths in the short and long term.

## Figures and Tables

**Table 1:** Descriptive statistics of variables.

Variable	Minimum	Maximum	Mean	Standard Deviation	25th Percentile	50th Percentile	75th Percentile
PMR men	284	720	485	92.9	415	486	534
PMR women	109	274	192	34.2	168	191	211
WS	61.7	99.99	88.1	9.86	81.4	89.1	96.7
SS	30.2	98.99	64.7	18.1	52.3	64	75.6
NO_x_	39.7	7820	898	1770	130	262	694
SO_2_	11.9	46,800	1290	5520	29.1	56.9	204
PM	107	3010	578	629	243	400	553
CO	168	113,000	6720	24,780	405	699	1260

Note: PMR—preventable mortality, WS—water supply, SS—sanitary sewer, NO_x_—nitrogen oxides, SO_2_—sulphur dioxide, PM—particular matter, CO—carbon monoxide.

**Table 2:** Estimated cross-sectional models.

Variable	Men	Women
Intercept	454.896 ***	138.550 **
Water Supply	2.036	0.973
Sanitary sewer	−2.878 ***	−0.715 **
SO2	−0.004 **	−0.001 *
Particular matter	0.072 *	0.024
CO	−0.002	−0.001
Least-developed	42.691	17.115
R-squared	0.397	0.164

Note: ***, **, * denote significance levels of 1, 5, and 10 percent, respectively. According to Breusch-Pagan test, there is no heteroscedasticity in the model for men (BP = 2.56), as well as in the model for women (BP = 1.49). Based on the Jarque-Bera Normality test, the assumption of residual normality is not violated for both sexes. For men, LM = 2.62, and for women, LM = 0.360.

**Table 3:** Optimal number of clusters.

Method	Silhouette	Duda-Hart Index	Milligan-Cooper Index	Ball-Hall Index	McClain-Rao Index
Number of clusters	3	3	3	3	3
Value Index	0.323	0.880	1890	114	0.528

**Table 4:** Centroids of clusters.

Cluster	Water Supply	Sanitary Sewer	NO_x_	SO_2_	Particular Matter	CO
Red	99	80	5990	27,600	2220	57,300
Green	93	70	439	228	362	1220
Blue	79	50	271	112	548	841

Note: NO_x_—nitrogen oxides, SO_2_—sulphur dioxide, CO—carbon monoxide.
